# Length of Detention Under the Mental Health Act at Farnham Road Hospital, Surrey (SABP)

**DOI:** 10.1192/bjo.2025.10640

**Published:** 2025-06-20

**Authors:** Mohsin Nazeer Muhammed, Pasupathy Tharmapathy, Lena Berger

**Affiliations:** 1 Surrey and Borders Partnership NHS Foundation Trust, Surrey, United Kingdom; 2 Frimley Health Foundation Trust, Frimley, United Kingdom

## Abstract

**Aims:** This audit aimed to evaluate the length of detention (LOD) for patients admitted to Farnham Road Hospital under the Mental Health Act (MHA), 1983. The objectives were to compare local LOD data with national figures and explore variations across age, gender, and ethnicity. This work aligns with the MHA Code of Practice’s focus on reducing restrictive practices and promoting equitable care.

**Methods:** This retrospective audit included 242 patients initially detained under the MHA across Surrey and Borders NHS Trust between 1 June and 30 November 2023. Local data was obtained from the trust MHA office. After applying inclusion criteria, 91 patients from four working-age adult wards at Farnham Road Hospital, Surrey were analysed. Patients detained under Sections 2 and 3 with lapsed, rescinded, or Community Treatment Order (CTO) outcomes were included. Data on age, gender, and ethnicity were analysed using ANOVA and t-tests for local comparisons, while national data from NHS Digital (2021–2022) were used to compare median LOD and interquartile ranges (IQR).

**Results:** The mean LOD for the local cohort was 38.4 days (SD=33.8). Of the 91 patients, 18–34-year-olds had a mean LOD of 34.9 days (SD=26.7), 35–49-year-olds 37.6 days (SD=35.4), and 50–64-year-olds 48.1 days (SD=46.4), with no significant differences across age groups (p=0.359). Male and female patients showed no significant differences in LOD locally (median = 27 days for both, IQR = 21–49 for males and 23–42 for females). Ethnicity comparisons showed disparities, with Black patients having a median LOD of 24 days and Asian patients 32.5 days locally, but limited sample sizes precluded statistical significance.

Nationally, the median LOD was shorter (18–34 years: 21 days, IQR = 6–38), increasing with age. Local figures were slightly higher for younger and middle-aged adults but similar for older adults. Ethnicity comparisons revealed greater variability locally, reflecting small sample sizes in minority groups. COVID-19 likely impacted national data more significantly, given the earlier timeframe (2021–2022).

**Conclusion:** This audit found no statistically significant differences in the length of detention (LOD) across age, gender, or ethnicity locally, suggesting equitable application of the Mental Health Act at Farnham Road Hospital. Median LODs were slightly higher locally compared with national data, but differences may reflect sample size, data collection periods, and local population characteristics. Expanding future audits to cover extended periods, aligning local and national data collection timelines, and obtaining detailed national datasets could enable more robust statistical analysis. Additionally, comparing pre- and post-COVID data and assessing other restrictive practices, such as physical and chemical restraints or seclusion, could provide a more comprehensive understanding of restrictive interventions and their impact on patient care.

